# Guillain-Barré syndrome: clinical profile and management

**DOI:** 10.3205/000220

**Published:** 2015-09-21

**Authors:** Sreenivasa Rao Sudulagunta, Mahesh Babu Sodalagunta, Mona Sepehrar, Hadi Khorram, Shiva Kumar Bangalore Raja, Shyamala Kothandapani, Zahra Noroozpour, Mohammed Aheta Sham, Nagendra Prasad, Sony Parethu Sunny, Munawar Dhanish Mohammed, Rekha Gangadharappa, Ranjitha Nidsale Sudarshan

**Affiliations:** 1 Columbia Asia Hospital, Bangalore, India; 2 K.S. Hegde Medical College, Mangalore, India; 3 Baptist Hospital, Bangalore, India; 4 Dr.B.R. Ambedkar Medical College, Otolaryngology Department, Bangalore, India; 5 Dr.B.R. Ambedkar Medical College, Bangalore, India

**Keywords:** Guillain-Barré syndrome, autoimmune, acute inflammatory demyelinating polyradiculoneuropathy, conduction motor action potential, IVIG, immunoglobulin, electromyography studies, plasma exchange

## Abstract

**Introduction:** Guillain-Barré syndrome (GBS) is a fulminant polyradiculoneuropathy that is acute, frequently severe and autoimmune in nature. Etiology of GBS is incompletely understood, prognosis is usually good with early detection and prompt treatment. This retrospective study was done to evaluate clinical profile, epidemiological, laboratory, and electrodiagnostic features of patients with GBS and mode of management, complications and prognostic factors.

**Methods:** Data of 1,166 patients admitted with GBS or presented to outpatient department (previous medical records) with GBS between January 2003 and January 2014 were analyzed.

**Results:** No difference in genders noted. Around 35% of patients are above 50 years of age. Poor control of diabetes with mean HbA_1c_ of 8.1 ± 2.11 is found on analysis. Seasonal occurrence in GBS is prominent in winter 484 (41.50%) and mechanically ventilated were 449 (38.50%) patients. 48 (4.11%) deaths were attributed to GBS. Neurological analysis revealed cranial nerve involvement in 407 (34.90%) patients, facial palsy in 401 (34.39%) and ataxia in 88 (7.54%) patients. Most patients in plasma exchange group belonged to the lower socio-economic status. Mean cerebrospinal fluid (CSF) protein levels was (n=962) 113.8 ± 11.8 mg/dl. Conduction block determined indirectly by absent H-reflex was noted in 891 (90.64%) patients. No difference in complications and outcome is found in treatment regimens of intravenous immunoglobulin (IVIG) and plasma exchange.

**Conclusion:** Seasonal occurrence predominantly in winter is noted. Peak flow test may be a predictor of assessing requirement of mechanical ventilation and prognosis. Conduction block is the major abnormality noted in electrophysiological studies and proximal nerve segment assessing with Erb’s point stimulation has high predictive value. IVIG treatment is more expensive but is associated with less duration of hospital stay.

## Zusammenfassung

**Einführung:** Das Guillain-Barré-Syndrom (GBS) ist eine fulminant verlaufende Polyradiculoneuropathie, die akut, meist schwer verlaufend, auf der Basis eines Autoimmunprozesses auftritt. Die Ätiologie der Erkrankung wird nicht ganz verstanden, die Prognose ist bei früher Diagnose und Therapie gewöhnlich gut. Eine retrospektive Studie wurde durchgeführt, um das klinische Profil, die Epidemiologie, die Laborwerte, die Elektrodiagnose, die Behandlungsarten und die Prognose von Patienten mit GBS auszuwerten.

**Methode:** Die klinischen Daten von 1.166 Patienten, die zwischen Januar 2003 und Januar 2014 mit GBS überwiesen oder in den Ambulanzen vorgestellt wurden, wurden ausgewertet.

**Ergebnisse:** Geschlechtsspezifische Unterschiede wurden nicht beobachtet. Etwa 35% der Patienten waren älter als 50 Jahre. Die Analyse zeigte schlecht eingestellten Diabetes mellitus (HBA_1_c = 8,1 ± 2,11%). Die saisonale Abhängigkeit der GBS ist deutlich, im Winter wurden 484 (41,5%) gefunden und 449 (38,5%) Patienten mit GBS wurden künstlich beatmet. 48 (4,11%) der Patienten verstarben an GBS. Die neurologischen Untersuchungen ergaben bei 407 (34,9%) der Patienten Beteiligung der cranialen Nerven, faciale Lähmungen bei 401 (34,39%) und Ataxien bei 88 (7,54%) der Patienten. Die meisten Patienten, die mit Plasmapherese behandelt wurden, hatten einen niedrigeren sozioökonomischen Status. Die mittlere Proteinkonzentration im Liquor war 113,8 ± 11,8 mg/dl. Störungen der Nervenleitung, indirekt bestimmt über den H-Reflex, wurden bei 891 (90,64%) der Patienten beobachtet. Keine Unterschiede hinsichtlich Komplikationen und Endergebnis wurden festgestellt zwischen den Behandlungen mit intravenöser Gabe von Immunglobulinen und Plasmaaustausch.

**Schlussfolgerung:** GBS tritt vorwiegend in den Wintermonaten auf, der „Peak-Flow-Test“ kann ein Indikator für eine erforderliche künstliche Beatmung und für die Prognose sein. Eine Verminderung oder Blockierung der Nervenleitung ist die schwerwiegendste Veränderung, die bei elektrophysiologischen Untersuchungen beobachtet wird. Die Prüfung des proximalen Nervensegments mit der Stimulierung am Erb-Punkt hat einen hohen prädiktiven Wert. Intravenöse Immunglobulin-Behandlung ist teurer, aber verkürzt den Krankenhausaufenthalt.

## Introduction

Guillain-Barré syndrome (GBS) is a fulminant polyradiculoneuropathy that is acute, frequently severe and autoimmune in nature. GBS is the most common cause of acute or subacute generalized paralysis which at one time rivaled polio in frequency [1]. GBS is also known as Landry-Guillain-Barré-Strohl syndrome and acute inflammatory demyelinating polyneuropathy (AIDP). Global annual incidence is reported to be 0.6–2.4 cases per 100,000 per year [2], [3], [4]. Men are more commonly affected by approximately 1.5 times than women [5]. Acute inflammatory demyelinating polyradiculoneuropathy (AIDP) is the most commonly occurring subtype in North America and Europe accounting for about 90% of all cases [6]. However, in other parts of the world (Asia, Central and South America) axonal variants of GBS i.e. acute motor axonopathy (AMAN) and acute motor sensory axonopathy (AMSAN) are found to represent 30% to 47% of cases [6], [7].

The earliest description of GBS dates to 19^th^ century regarding an afebrile generalized paralysis by Wardrop and Ollivier in 1834. Other important landmarks are Landry’s report in 1859 about an acute, ascending, predominantly motor paralysis with respiratory failure, leading to death [8] and Osler’s (1892) description of afebrile polyneuritis [9]. Guillain, Barré, and Strohl (1916) described a benign polyneuritis with albumino-cytological dissociation in the cerebrospinal fluid (CSF) [10] and the first report regarding pathology of GBS was by Haymaker and Kernohan in 1949 who reported that edema of the nerve roots was an important change in the early stages of the disease [11]. Asbury, Arnason and Adams (1969) established that the essential lesion is due to perivascular mononuclear inflammatory infiltration of the roots and nerves [12].

Etiology of GBS is not completely understood but believed to be due to autoimmune cause where majority of cases are triggered by infection stimulating anti-ganglioside antibodies production. Approximately 70% of cases of GBS occur 1–3 weeks after an acute infectious process. The organisms thought to be involved are *Campylobacter jejuni* (diarrhea), *Mycoplasma pneumonia*, *Haemophilus influenzae*, cytomegalovirus, Epstein-Barr virus and influenza [13]. Administration of outmoded anti-rabies vaccines and A/New Jersey (swine) influenza vaccine, given in 1976, was associated with a slight increase in GBS incidence. New influenza vaccines appear to confer risk of <1 per million and are relatively safe. 

Clinical features include areflexia, limb weakness and uncommonly sensory loss proceeding to neuromuscular paralysis involving bulbar, facial and respiratory function with maximum severity of symptoms in 2–4 weeks [13]. Common clinical pattern is an ascending paralysis first noticed as rubbery legs which evolves over hours to few days with tingling and dysesthesias in the extremities frequently accompanying. Lower limbs are affected more than upper limbs, and facial diparesis occurs in 50% of affected individuals. Usually prognosis is good with 90% of patients having complete functional recovery or with minimal deficits in 1 year after the onset of GBS [3], [14]. Mortality rate is between 1–18% [15]. Diagnosis of AIDP is made by recognizing the pattern of paralysis that is rapidly evolving along with areflexia, absence of fever or other systemic symptoms (Table 1 (Tab. 1)) [16]. 

The seasons in the Indian subcontinent were divided as: summer/pre-monsoon: March to May; monsoon/rainy: June to September; post-monsoon/autumn: October to November; winter: December to February according to the seasonal classification of the Indian Meteorological Department. This retrospective study which evaluated 1,166 patients was conducted to study clinical profile, epidemiological, laboratory, and electrodiagnostic features of patients with GBS and mode of management, complications and prognostic factors.

## Methods

The retrospective study analyzed data of patients admitted with GBS or presented to outpatient department (previous medical records) with GBS between January 2003 and January 2014. Data was pooled from 7 hospitals in which 4 are specialized neurological centers. The medical records were analyzed for the demographic data (age, sex), clinical features, co-morbid conditions, investigations, electrophysiological data, mode and results of the treatment and complications of the procedures. 1,166 patients fulfilled the levels 1, 2 or 3 described by Sejvar et al. which are of diagnostic certainty for GBS/MFS [17].

Medical Research Council (MRC) sum score was used for valuing the muscle strength from 0 to 5 in proximal and distal muscles in upper and lower limbs bilaterally; score ranged from 40 (normal) to 0 (quadriplegic) and by Hughes et al. disability score for GBS [18]. Cranial nerve involvement was noted along with respiratory muscle weakness which was assessed by need of mechanical ventilation, oxygen administration, non-invasive ventilation and spirometer record. Sensory system and autonomic abnormalities were analyzed. 

Classification of patients as axonal or demyelinating subtype was based on electrodiagnostic criteria of Hadden et al. [19]. AMSAN diagnosis was based on criteria of Rees et al. [20]. CSF examination was available in 884 (75.81%) patients. Even though 1,166 patient records were analyzed for demographic data, specific treatment protocols with plasma exchange or intravenous immunoglobulin were available in 962 patients and were analyzed for mode of treatment and complications.

Records indicated that serological testing was done for cytomegalovirus (CMV), herpes simplex virus (HSV), Epstein-Barr virus (EBV), varicella-zoster (VZV), *Mycoplasma pneumoniae*, hepatitis B and C, *Haemophilus influenzae* and *Campylobacter jejuni*. GBS disability score [18] (Table 2 (Tab. 2)) was used for evaluation of functional impact during discharge of patients.

962 patients received treatment with either IVIG or plasma exchange, of which 494 (51.35%) received IVIG and 468 (48.64%) plasma exchange. Plasma exchange regimen is considered as removal of 200–250 mL/kg of plasma (total) over 5 to 8 cycles on a daily or alternate day basis. Plasma exchange was performed using a peripheral or central venous catheter. Replacement fluids used were fresh frozen plasma (FFP), lactated Ringer’s solution (RL), substitute fluid (SF) and 3% hydroxylethyl starch (HES). TPE was performed every alternate day for majority of patients and 1 plasma volume was exchanged during each procedure. IVIG was administered as 0.4 g/kg daily for 5 consecutive days.

The common indigenous IVIG preparations available in India are Bharat Serum: Ivigama, VHB Pharma: Iviglob, Intas: Globucel, Reliance: Immunorel, Claris: Norglobin, Synergy: MeGlob, and Nirlife Healthcare: IVIG. FDA or EMA preparations of IVIG available in India are Biotest: Intratect, Intraglobin, and Pentaglobin, Bharat Serum/Talecris: Gamunex, Baxter: Kiovig, Gammagard, Novartis: Sandoglobulin, Octapharma: Octagam, and Alpha: Venoglobulin. The average cost per 5 grams of indigenous preparations is around INR 7,500 (US $ 138) while FDA or EMA approved preparations cost around INR 25,000 (US $ 462).

Statistical analyses were performed using SPSS 16 program. Chi-square test for dichotomic variables was used for univariate analysis. Student t-test in parametric variables was used for continuous variables. Mean, standard deviation was performed. P-value was considered significant if <0.05. For calculation of treatment costs, Indian rupee (INR) to US dollar (USD) conversion is done as per standard conversion rates on January 1^st^ 2015. 

## Results

**Figure 1:**
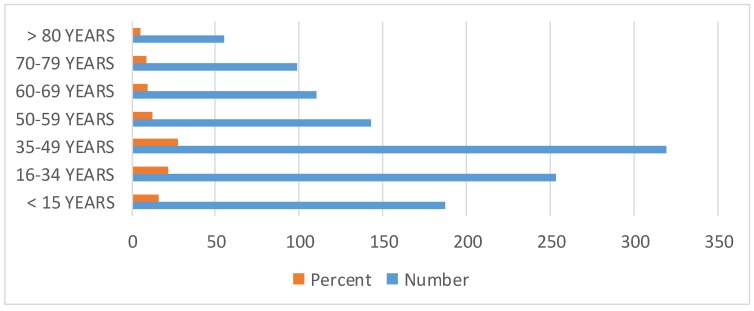
Bar diagram showing age wise distribution

**Figure 2:**
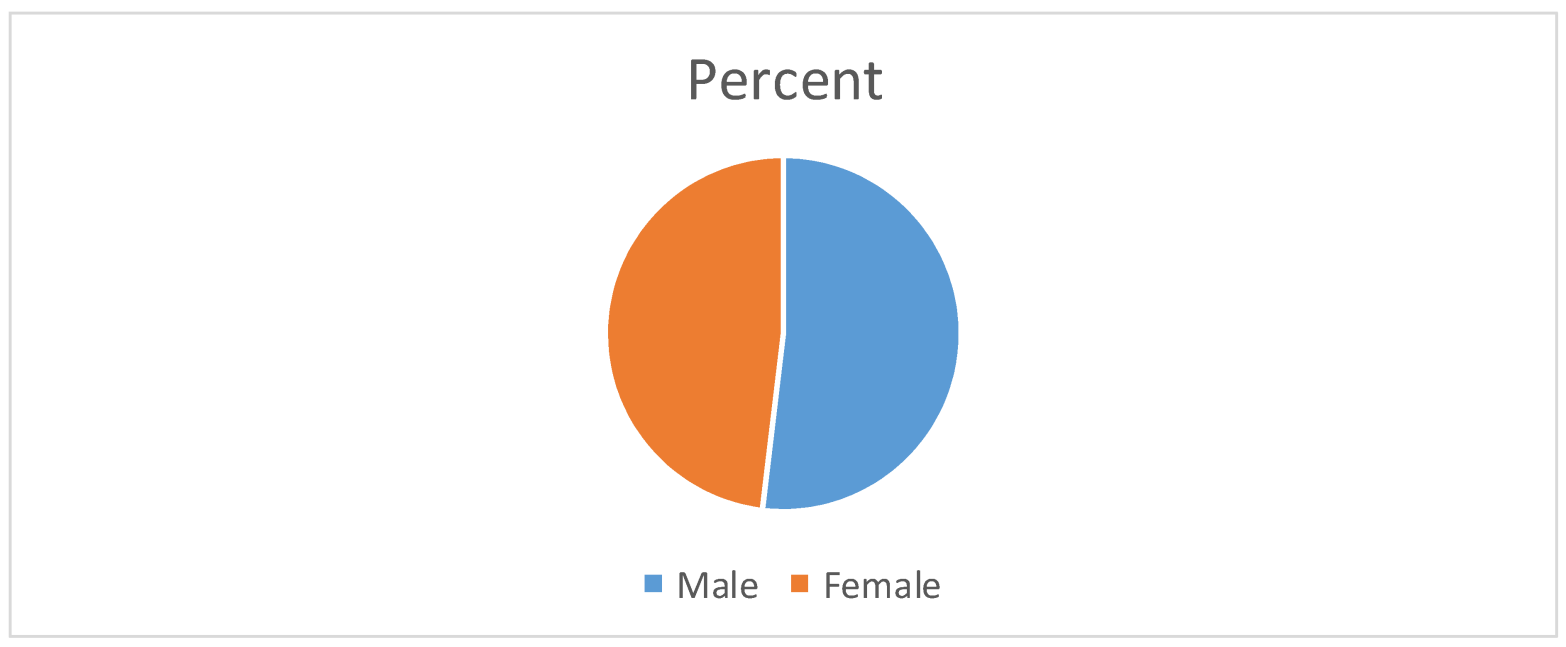
Pie chart showing gender distribution

**Figure 3:**
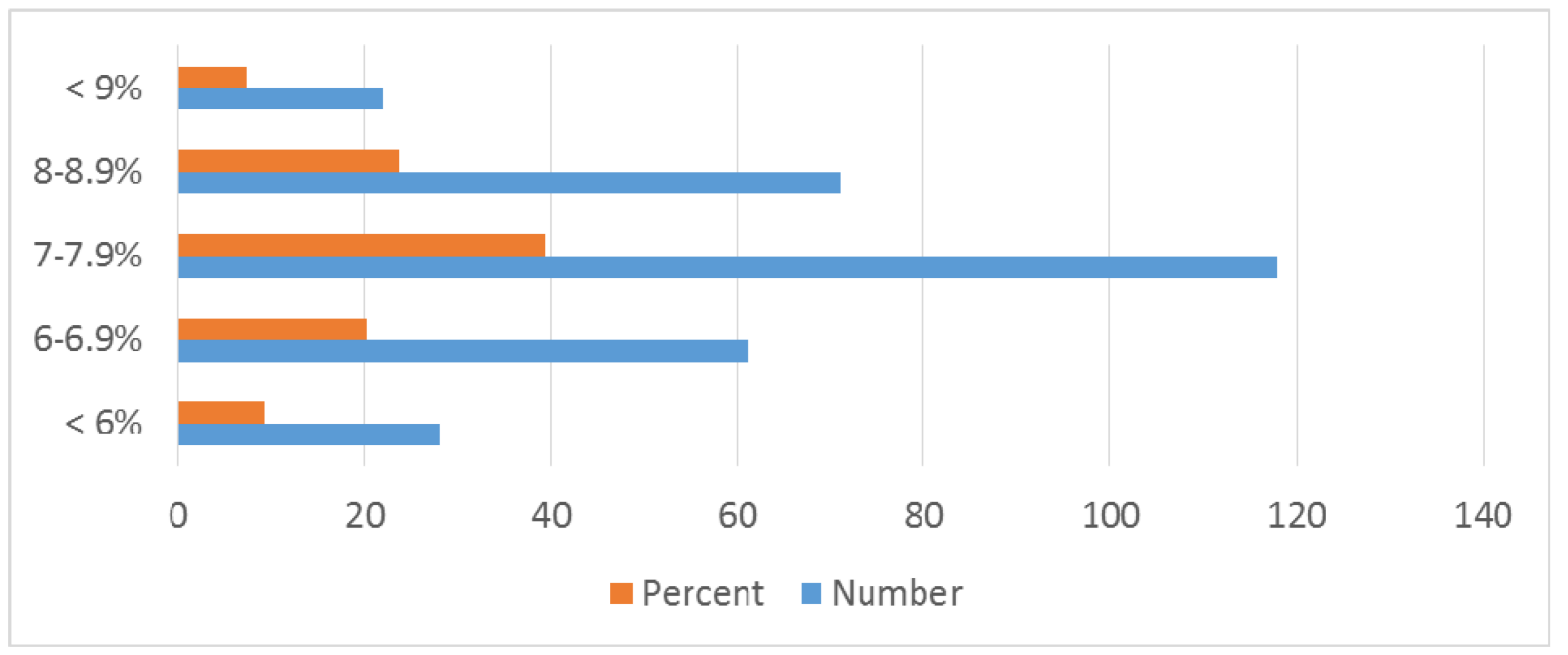
Bar diagram showing HbA_1c_ distribution

**Figure 4:**
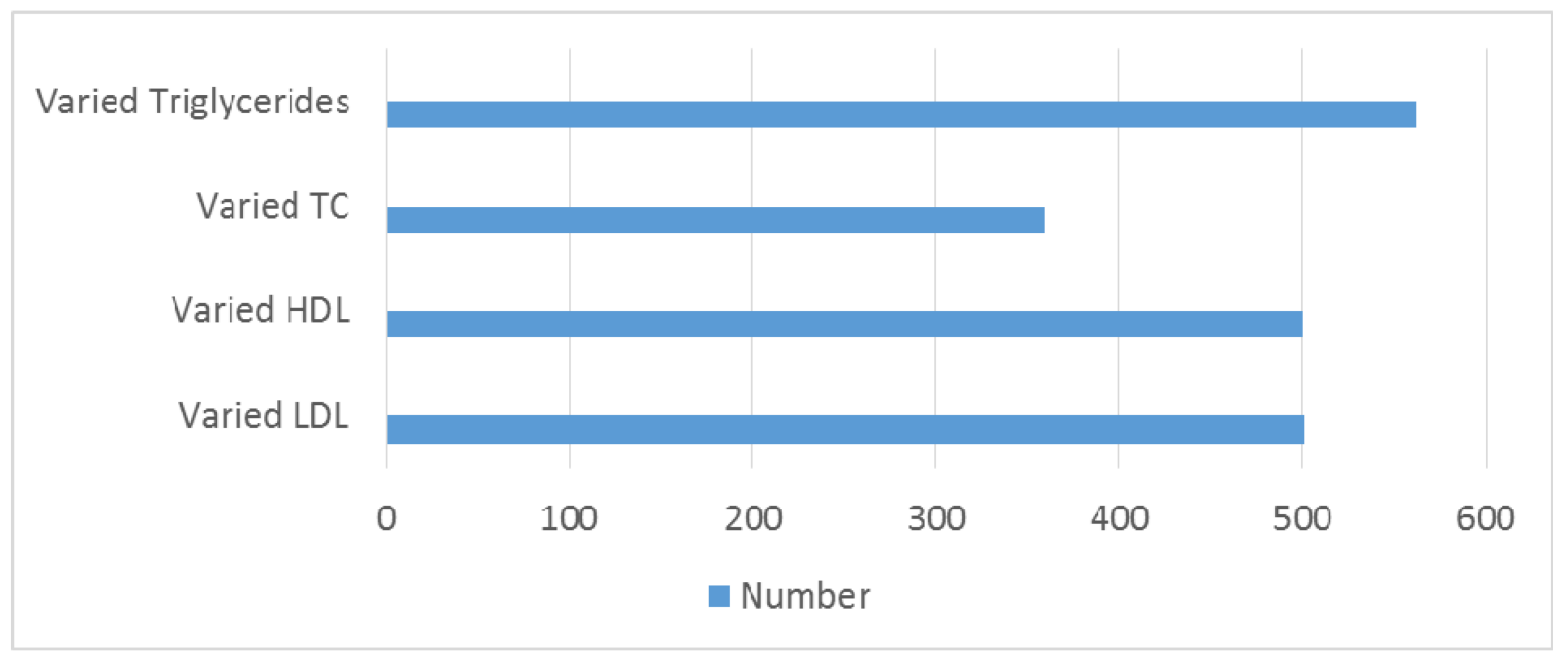
Bar diagram showing lipid parameters

Study group of 1,166 patients consisted of 605 (51.88%) males and 561 females (48.11%). Mean age of onset was 42.8 ± 24 years (range from 0–85). Demographic and clinical data are illustrated in Table 3 (Tab. 3) and in Figure 1 (Fig. 1), Figure 2 (Fig. 2), Figure 3 (Fig. 3), Figure 4 (Fig. 4). There is no difference in genders. Around 35% of patients are above 50 years of age. Diabetic patients constituted 400 out of 1,166 patients. Poor control of diabetes with mean HbA1c of 8.1±2.11 is found on analysis. 70.33% of diabetic patients had HbA_1c_ >7%. Even though dyslipidemia is found in 70% of patients only 16% patients are taking statins which is alarming (Table 3 (Tab. 3)).

**Figure 5:**
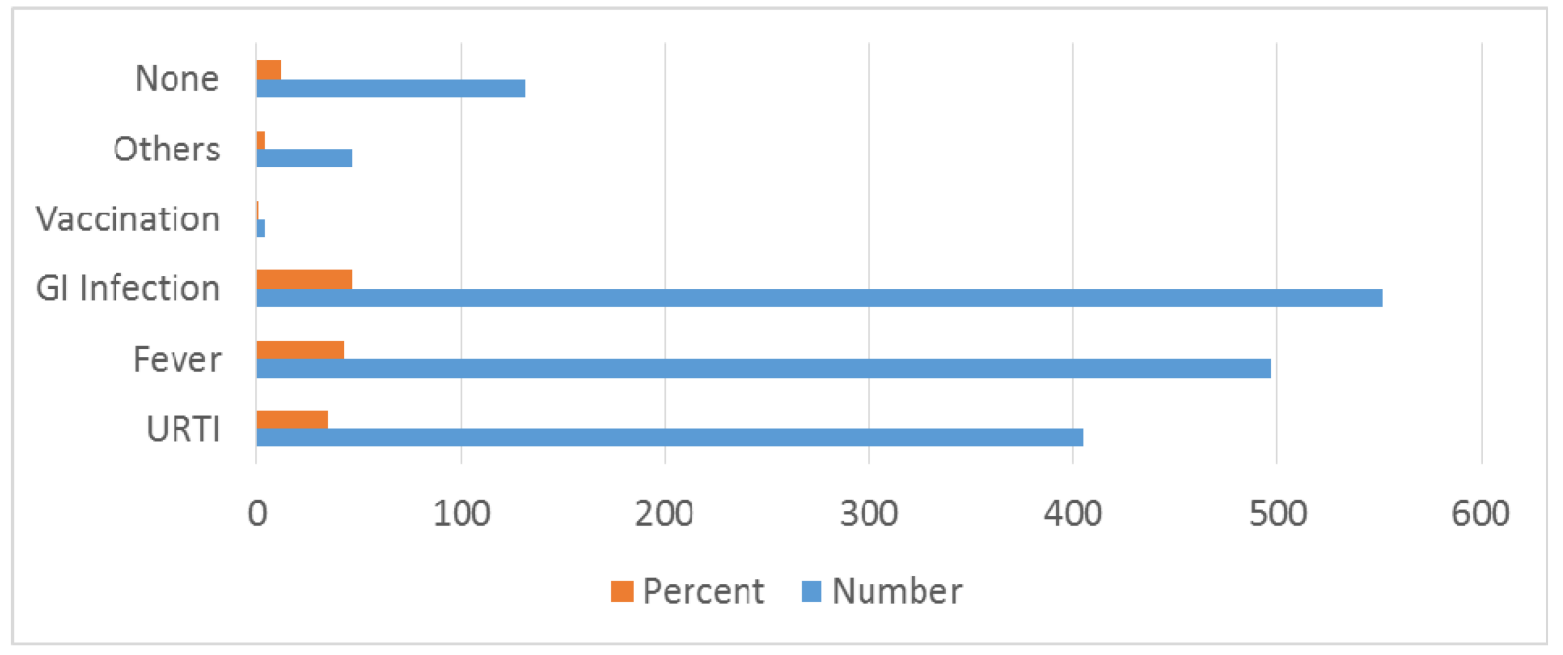
Bar diagram showing antecedent events

**Figure 6:**
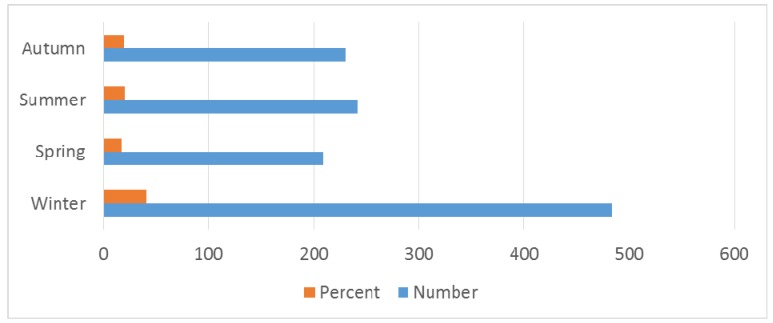
Bar diagram showing seasonal occurrence

**Figure 7:**
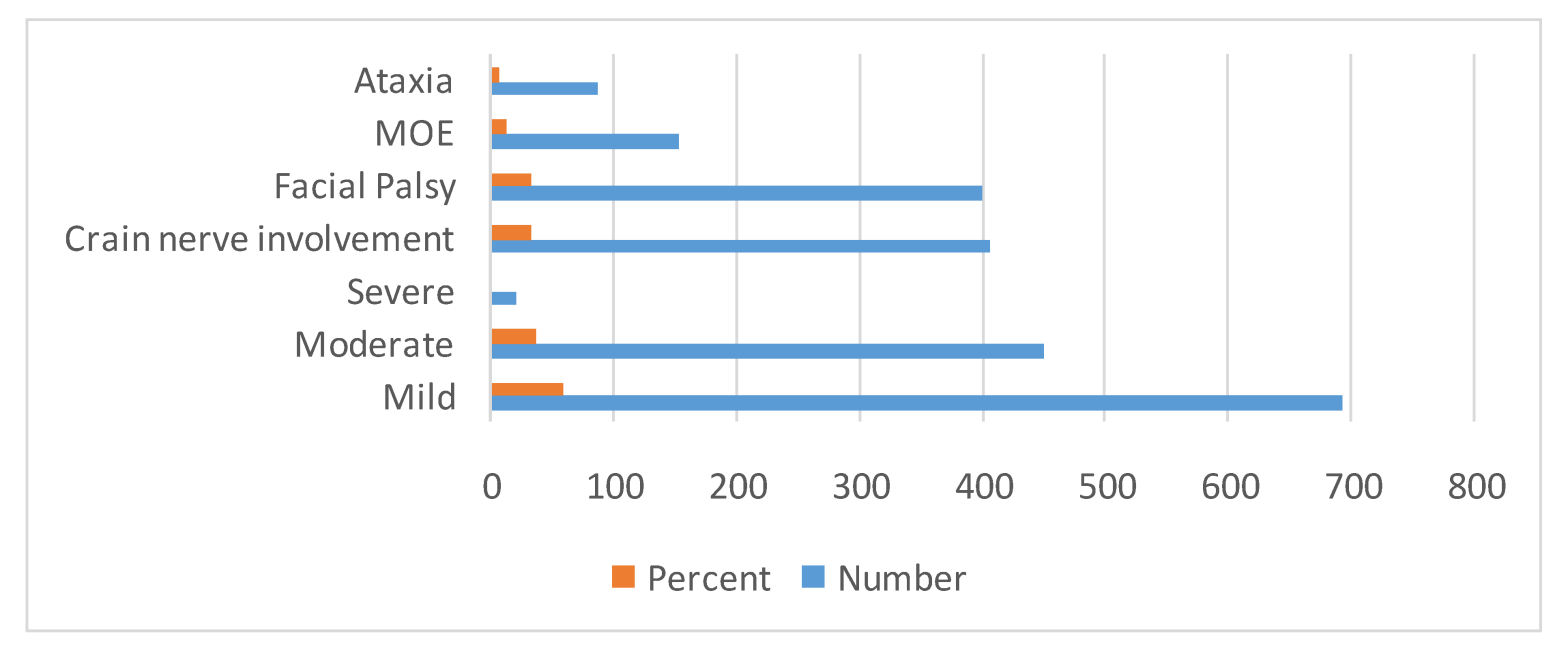
Bar diagram showing motor deficits

**Figure 8:**
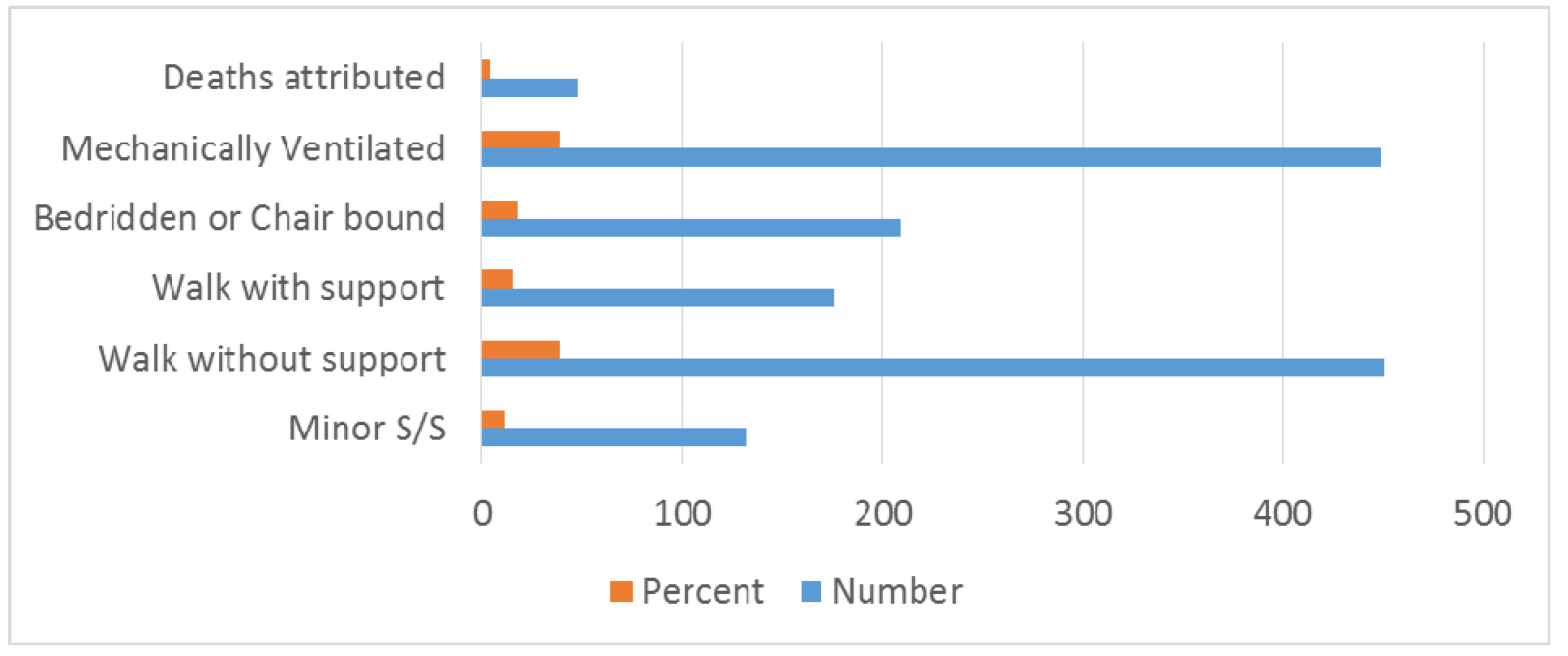
Bar diagram showing GBS disability score

Among antecedent events that were associated with GBS, fever was present in 497 (42.62%) patients and gastrointestinal infection in 551 (47.25%) patients. Seasonal occurrence in GBS is prominent in winter 484 (41.50%), monsoon/rainy season 209 (17.92%) and summer/pre-monsoon 242 (20.75%) seasons (Table 4 (Tab. 4), Figure 5 (Fig. 5), Figure 6 (Fig. 6)). Neurological deficits and GBS disability score are illustrated in Table 5 (Tab. 5) (Figure 7 (Fig. 7), Figure 8 (Fig. 8)). According to GBS disability score, minor signs or symptoms were found in 132 (11.32%) patients, 451 (38.67%) patients walked without support, 176 (15.09%) walked with support, 209 (17.92%) were bedridden or chair bound and 449 (38.50%) patients were mechanically ventilated. 48 (4.11%) deaths were attributed to GBS. 

Neurological analysis revealed cranial nerve involvement in 407 (34.90%) patients, facial palsy in 401 (34.39%) and ataxia in 88 (7.54%) patients (Table 5 (Tab. 5)). Electrodiagnostic features (abnormal frequencies) in Guillain-Barré syndrome patients are illustrated in Table 6 (Tab. 6). The highest frequency of affection in patients was observed in the sixth (36.7%) and fifth (27.9%) decade of life. In patients classified as axonal variety, electrophysiology showed low distal CMAP amplitudes, normal motor, sensory conduction velocities and normal distal latencies. F waves were absent or of normal latency and normal sensory neurography. 891 (90.64%) patients had absent H-reflex in soleus muscle. Conduction block (CB) in Erb-to-axilla segment was noted in 503 (78.59%) of 962 patients. 

CMAP amplitude less than 3 mV was noted in the thumb abductor muscle in 183 (28.59%) of 640 patients and in little finger abductor muscle in 137 (21.40%) of 640 patients. But, amplitude reductions observed were not accompanied by CMAP duration prolongation. In the Erb to axilla segment compared to reduction of distal CMAP amplitude and conduction block in forearm (p=0.0004) and upper arm (p=0.0002), median nerve conduction block was more commonly involved and is statistically significant (p=0.0003). In the Erb-to-axilla segment compared to upper arm (p=0.0001), forearm (p=0.0001) and elbow (p=0.0031) segments, ulnar nerve conduction block involvement was common and statistically significant.

Motor conduction velocity delay is observed more commonly in Erb-to-axilla segment than in the upper arm (p=0.005) and forearm (p=0.0002) segments. Statistically significant difference was not observed between slowing of motor conduction velocity in the Erb-to-axilla segment and prolongation of F wave latency (p>0.05). The difference between patients who had normal sural nerve neurography and had normal median nerve sensory neurography was not statistically significant (p>0.05). There is statistically significant difference observed between reduction of sural nerve and median nerve sensory nerve action potential (SNAP) amplitudes compared to sensory conduction slowing down in both nerves (p=0.0001).

In the 962 patients where treatment records were available, 494 (51.35%) received IVIG and 468 (48.64%) plasma exchange. Males constituted 286 (57.89%) and 312 (66.66%) in IVIG and plasma exchange groups, while females constituted 208 (42.10%) and 156 (33.33%) in IVIG and plasma exchange groups. Mean and range of age were comparable in both groups. Clinical features at admission were almost similar in both groups. Limb weakness was observed in all patients. Distal weakness was more common than proximal weakness. It is the commonest clinical presentation observed. Epidemiological data of patients treated with IVIG or plasma exchange (962 patients) is illustrated in Table 7 (Tab. 7). 

AIDP was observed to be the commonest type followed by AMAN and AMSAN. CSF examination evaluation revealed mean CSF protein level of 113.8 ± 11.8 mg/dl (range: 18–450 mg/ dl). Plasma exchange group patients had statistically significant increase in mean length of stay compared to IVIG group (p=0.001) (Table 7 (Tab. 7)). Clinical features of patients that received treatment (962 patients) are illustrated in Table 8 (Tab. 8). Complications and outcome of GBS are illustrated in Table 9 (Tab. 9). There was no statistically significant difference of complication occurrence in both groups. Clinical outcome analysis showed statistically significant mean MRC sum score at time of admission and at time of discharge in IVIG group, which were 19.62 ± 8.20 and 40.10 ± 14.80 (p<0.0001); and in plasma exchange group were 19.42 ± 11.16 and 40.64 ± 15.80 (p<0.0001) respectively. 

**Figure 9:**
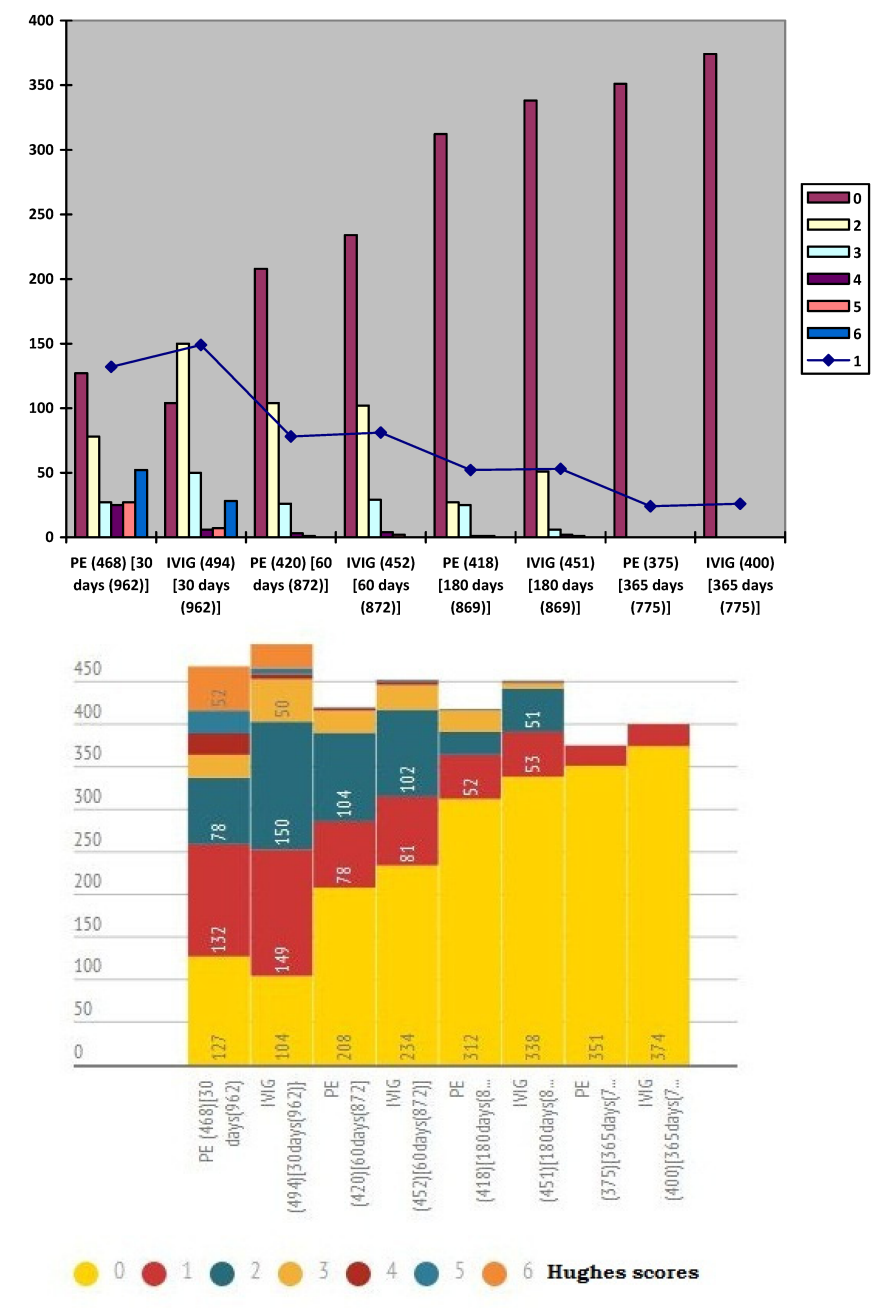
Follow-up of patients with GBS with Hughes score

Follow-up of patients with GBS were illustrated in Table 10 (Tab. 10) and Figure 9 (Fig. 9). At 30 days of follow-up, Hughes grade of 0 was observed in 127 (27.13%) patients of plasma exchange group and 104 (21.05%) patients of IVIG groups. At 180 days of follow-up, Hughes grade of 0 was noted in 312 (74.64%) patients of plasma exchange group and 338 (74.94%) patients of IVIG groups. At 365 days of follow-up, progressive improvement in muscle strength is noted. Hughes grade of 0 was noted in 351 (93.6%) patients of plasma exchange group and 374 (93.5%) patients of IVIG groups at 365 days follow-up. No statistically significant difference in follow-up and outcome at discharge was found at 30, 60, 180 and 365 days between both groups.

However, not all patients had financial records showing cost of hospital care available. The cost varied from government and private hospitals significantly. One important observation was that plasma exchange costs were significantly less than IVIG in all the hospitals (p<0.01). The cost of hospital care of the 2 groups is illustrated in Table 11 (Tab. 11).

## Discussion

Our retrospective study shows that there is no difference in gender similar to other studies [21]. Linear increase in incidence with age until 5th decade is noted and patients above 50 years constituted 35% of all patients [22], [23]. Winter predominance (484 (41.50%) patients) of occurrence of illness was noted in our study [23] followed by summer/pre-monsoon (242 (20.75%) patients) even though it was considered that GBS is sporadic without seasonal preference [13], [22]. Our observation differs from studies by Kaur et al. [24], Sharma et al. who reported a peak incidence between June–July and Sept–October [25] and Sriganesh K et al. in 2013, who reported increased occurrence of GBS during the months of June to August and December to February [26]. In many studies, patients admitted with predisposing or associated infection constituted 40–70% of patients [2], [22], [27]. Our study reveals that around 80% of patients had predisposing infection. Gastrointestinal infection was present in 551 (47.25%) followed by upper respiratory infection in 405 (34.73%) patients. 

Patients with syndrome of inappropriate antidiuretic hormone hypersecretion (SIADH) constituted 154 (13.20%) patients. In some studies SIADH was reported to be up to 58% in GBS. There is no clear consensus regarding the neurophysiological values defining Guillain-Barré syndrome and its variants [28], [29], [30], [31]. Motor conduction velocity (MCV) decrease, prolongation of motor distal latency, conduction blocks (CB), temporal dispersion and increased F wave latency are the commonly accepted demyelination parameters. First electromyography changes reported are the alteration of F wave and H-reflex response. The important electrophysiological features of peripheral nerve demyelination are conduction block and conduction slowing. In a study reported in 1981 regarding experimental demyelination, conduction block is an early manifestation of demyelination and slow conduction velocity is a feature of remyelination [32]. 

Many studies revealed that the main cause of acute paralysis and the most common observation in early GBS is conduction block and it may be the only sign in early GBS [33], [34]. This is also found in our study showing that conduction block is the commonest abnormality observed. Conduction block determined indirectly by absent H-reflex was noted in 891 (90.64%) patients, which was the most frequent electrophysiological abnormality observed in GBS patients. Many series of GBS revealed that electrophysiological abnormalities are unevenly distributed with more frequency of occurrence in most proximal and terminal segments of the peripheral nervous system and across entrapment sites [35], [36], [37], [38]. Relative deficiency of blood-nerve barrier may be reason for uneven distribution as per studies [39]. 

Our study shows concordance with previous studies in the distribution of electrophysiological abnormalities. Conduction block and motor conduction slowing were more frequent in the Erb-to-axilla segment than in distal segments which shows that proximal segment involvement was more common. Mills and Murray reported that the predominant abnormality in early GBS is proximal conduction block, which was measured directly in the nerve segment between axilla and spinal cord [40]. There was difference in conduction block frequency noted in carpal tunnel and elbow segments compared to forearm and upper arm even though it was not significant (p>0.05).

Mean CSF protein levels were increased in all the patients (n=962). 113.8 ± 11.8 mg/dl was the mean noted for CSF and is comparable to studies by Chiò et al. [41] and Corston et al. [42]. Studies reported that abnormal rise of CSF protein in GBS may be due to inflammatory reaction in the choroid plexus or disturbance in process of transport [11], [43], [44], [45], [46] or breakdown of the blood CSF barrier [47], [48], [49], [50], [51], [52]. 

Outcomes of patients in IVIG and plasma exchange groups were comparable, which were analyzed by muscle strength and Hughes grade. Our study is in concordance with other studies in outcomes [53], [54]. Few studies reported that plasmapheresis is better than IVIG in children with GBS under mechanical ventilation [55]. However, our study did not show any concordance. 

Frequencies of occurrence of complications were similar in both groups even though there are reports of increased risk of hypotension and spread of infection in plasma exchange group [56], [57]. 44 patients in plasma exchange group were reported to have breathing difficulty of which 40 of them improved over a period of 3–4 days. One specific incidence of transfusion related acute lung injury (TRALI) was reported in a female patient immediately after plasma exchange. However the patient was also simultaneously diagnosed with scrub typhus infection by Weil-Felix and antibody tests. Scrub typhus is a common infection usually underdiagnosed in rural Bangalore areas. Patient improved over a period of 7 days. The exact reason could not be found from the patient records. 

Another significant observation was that most patients in plasma exchange group belonged to the lower socio-economic status as per modified Kuppuswamy’s socio-economic scale used in India [58]. Also statistically significant longer hospital stay was noted in plasma exchange group compared to IVIG group. This may be due to the financial constraints of patients in plasma exchange group which may include delay in getting investigations and treatment. Deaths observed were 24 (4.85%) patients in IVIG group and 20 (4.27%) patients in plasma exchange group, slightly more than in studies reported from Europe and North America [59]. Even though IVIG is comparatively easier to administer and duration of hospital stay is lower compared to plasma exchange, it is more expensive. No difference was noted in the effectiveness of treatment or improvement rate by usage of either treatment. 

## Conclusion

We put forward the following observations based on our retrospective analysis. This retrospective study has the limitations in accurate calculation of incidence. No gender difference is observed. Increased age is associated with worse prognosis and increased frequency of GBS occurrence. Seasonal occurrence predominantly in winter is noted. Peak flow test may be a predictor of assessing requirement of mechanical ventilation and prognosis. Conduction block is the major abnormality noted in electrophysiological studies and proximal nerve segment assessing with Erb’s point stimulation has high predictive value. Proximal segment involvement is more common than distal segment involvement as per electrophysiology studies. 

No difference in complications and outcome is found in treatment regimens of IVIG and plasma exchange. Mortality rate is comparable. IVIG treatment is more expensive but is associated with less duration of hospital stay. 

## Abbreviations

- AIDP: Acute inflammatory demyelinating polyradiculoneuropathy
- AMAN: Acute motor axonal neuropathy
- AMSAN: Acute motor and sensory axonal neuropathy
- CB: Conduction blocks
- CMAP: Conduction motor action potential
- CMV: Cytomegalovirus
- CSF: Cerebrospinal fluid
- EBV: Epstein-Barr virus
- EMA: European Medicines Agency
- EMG: Electromyography studies
- GBS: Guillain-Barré syndrome
- HSV: Herpes simplex virus
- IVIG: Intravenous immunoglobulin
- MCV: Motor conduction velocities
- MFS: Miller-Fisher syndrome
- MRC: Medical Research Council
- MV: Mechanical ventilation
- NCS: Nerve conduction studies
- SIADH: Syndrome of inappropriate antidiuretic hormone hypersecretion
- SNAP: Sensory nerve action potentials
- VZV: Varicella-zoster virus

## Notes

### Competing interests

The authors declare that they have no competing interests.

## Figures and Tables

**Table 1:**
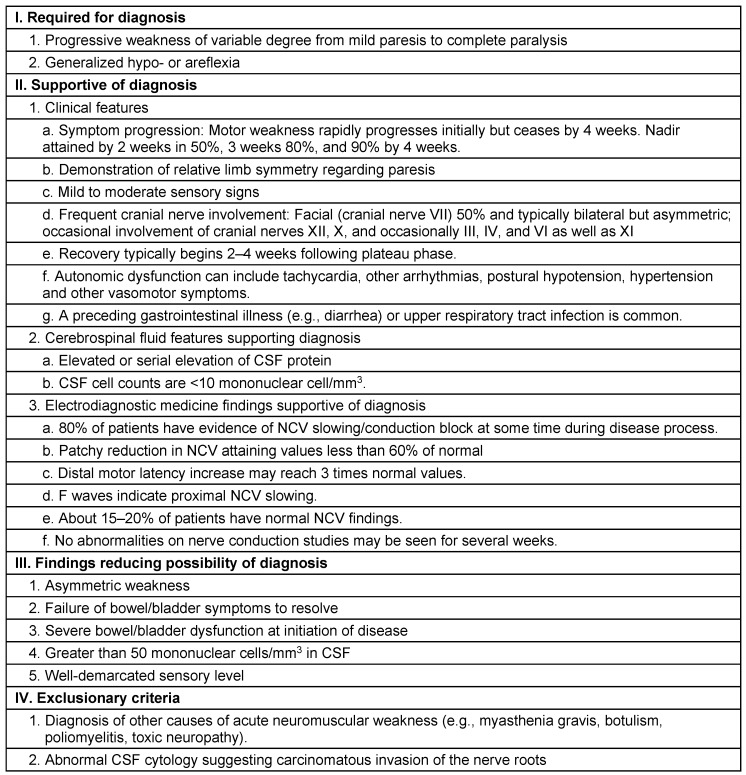
Diagnostic features of acute inflammatory demyelinating polyneuropathy (AIDP)

**Table 2:**
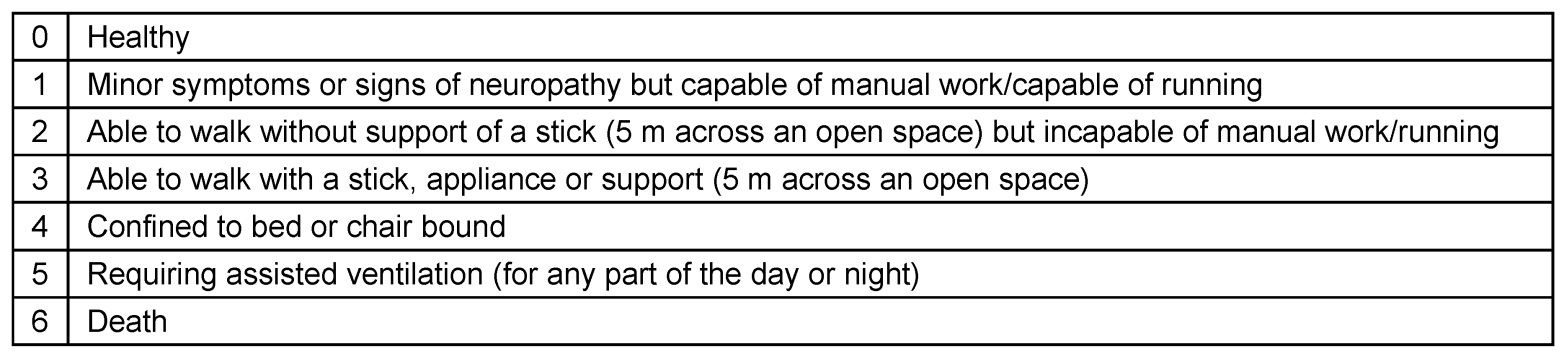
Hughes grade scale for assessing functional motor deficits

**Table 3:**
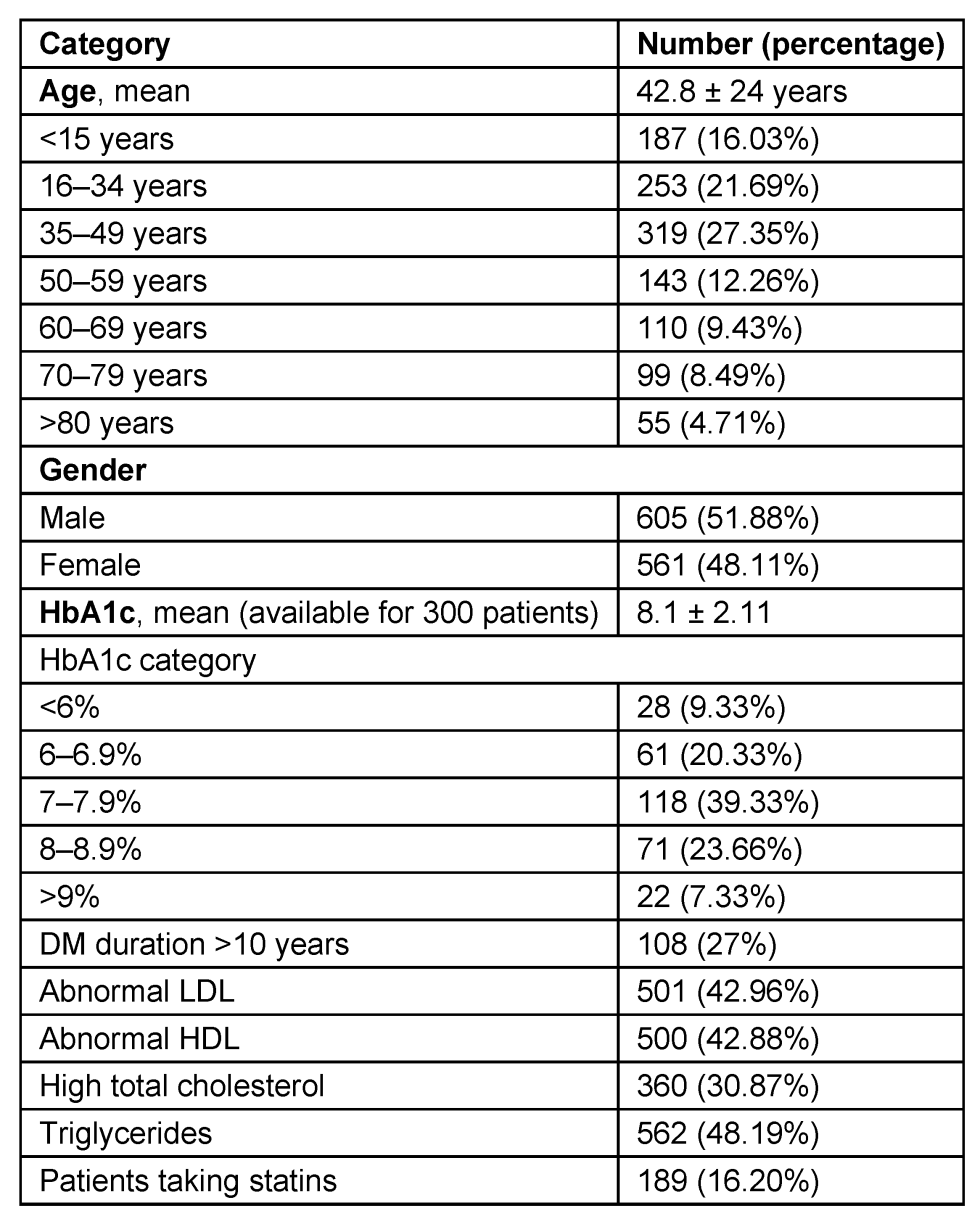
Epidemiological data of 1,166 patients

**Table 4:**
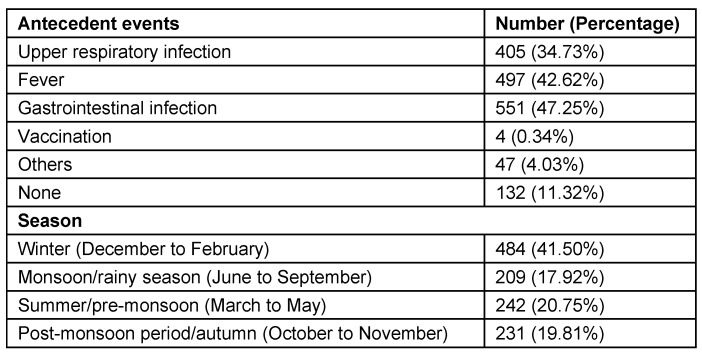
Seasonal occurrence and antecedent events in GBS

**Table 5:**
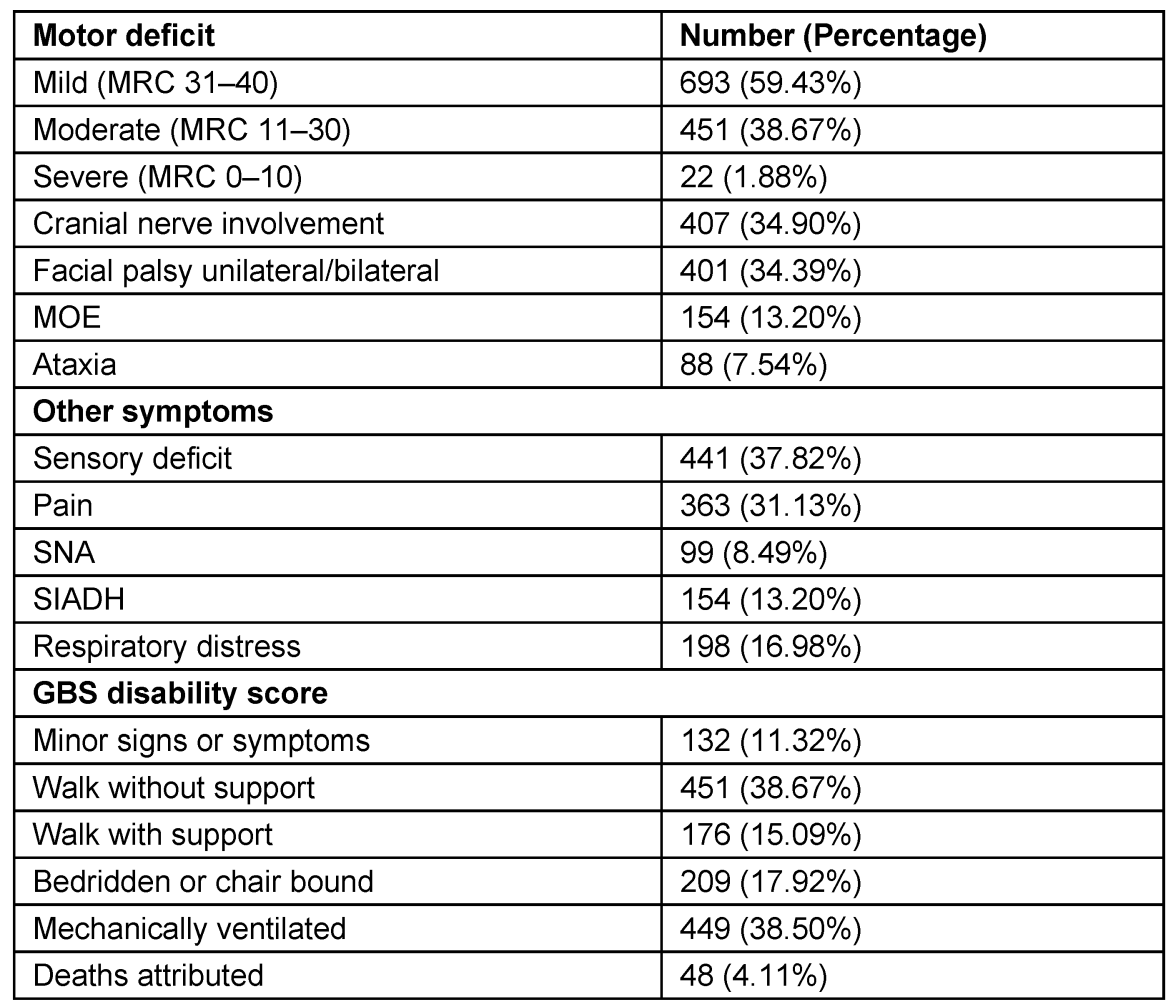
Neurological deficits and GBS disability score (1,166 patients)

**Table 6:**
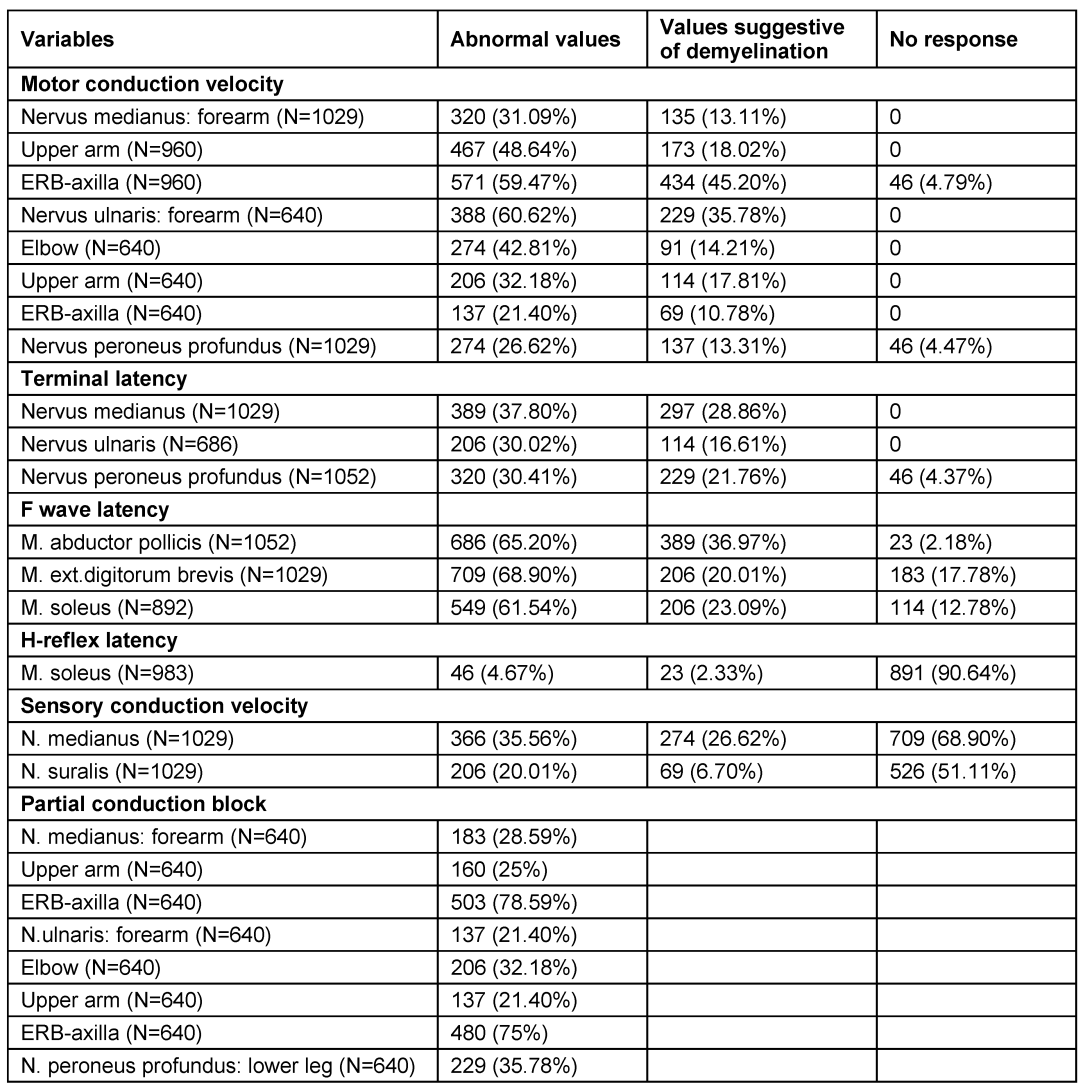
Electrodiagnostic features (abnormal frequencies) in Guillain-Barré syndrome patients (1,166 patients)

**Table 7:**
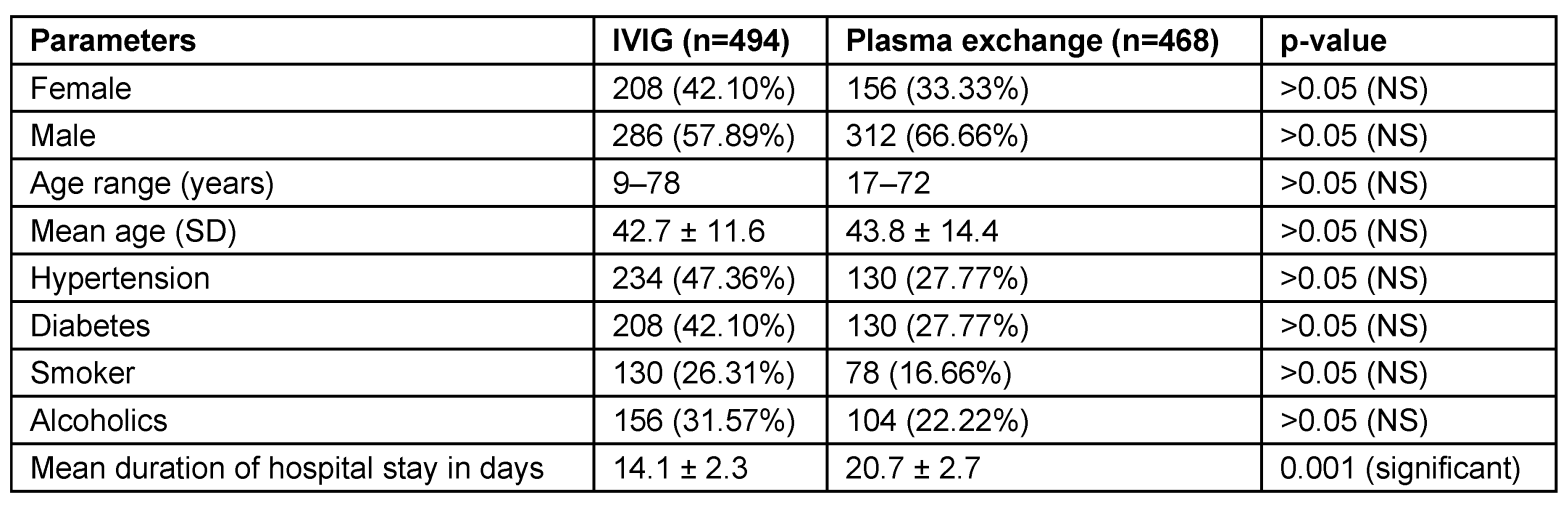
Epidemiological data of patients treated with IVIG or plasma exchange (962 patients)

**Table 8:**
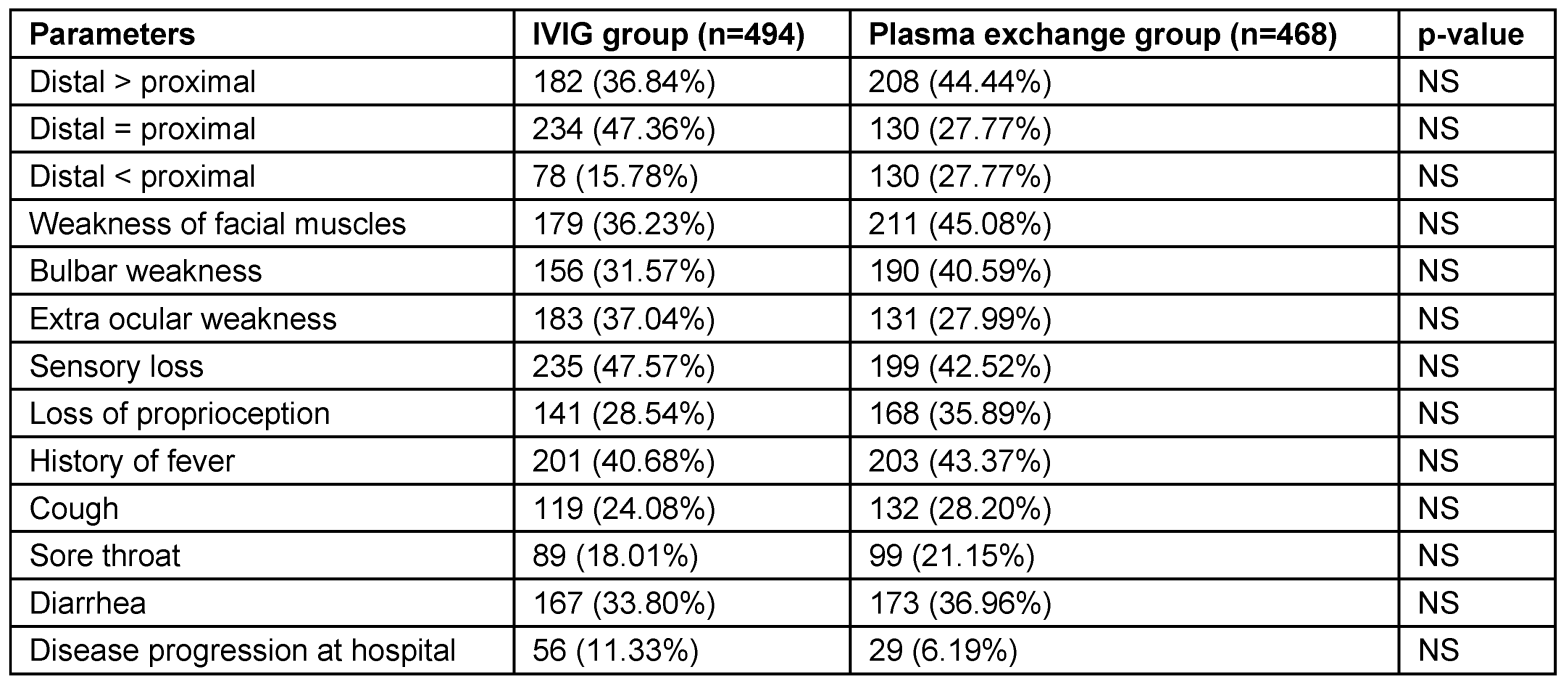
Clinical features of patients that received treatment (962 patients)

**Table 9:**
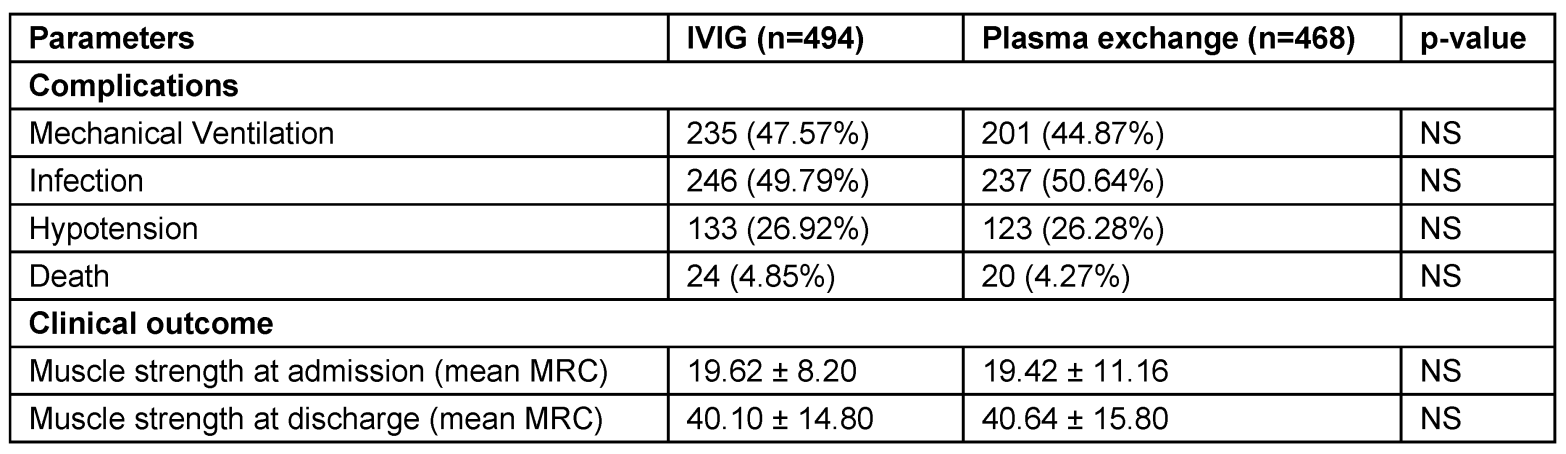
Complications and outcome of GBS

**Table 10:**
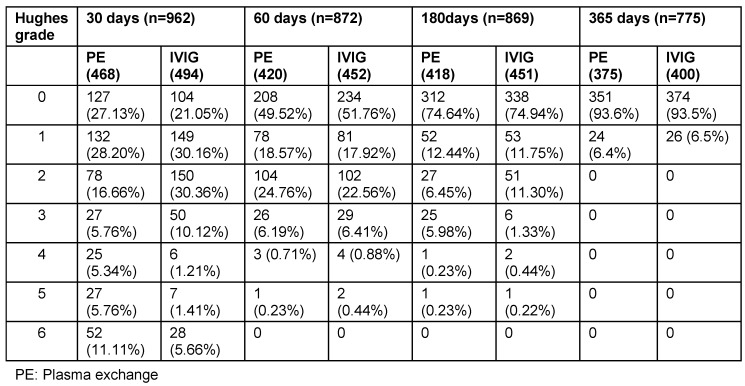
Follow-up of patients with GBS

**Table 11:**
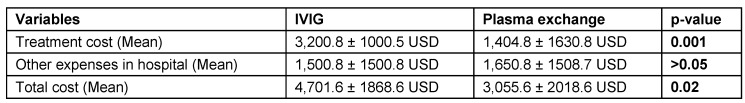
Hospital care cost in plasma exchange and IVIG groups
